# Treatise on the Generation of Intestinal Worms, and on the Means of Destroying Them

**Published:** 1785

**Authors:** 


II. *Abhandlung von der erzeugung der eingeweid-ewurmer und der mitteln wider dieselben. i. e. Treatife on the generation of inteftinal worms, and on the means of deftroying them. By* M. E. Bloch, *M. D. Phyfician at Berlin, Member of the Societies of Natural Hiftorians of Berlin, Dantzick, Halle, &c.* 4to. Berlin, 1782. 51 pages, with ten copper-plates.

THE Royal Society of Sciences at Copenhagen having offered a premium for the beft differtation on the origin of inteftinal worms, and the means of deftroying them, the prize was adjudged to the author of the treatife now before us.

The work is divided into three fections. In the firft fection, the author prefents us with a fyftematic arrangement of inteftinal worms; in in the fecond, he treats of their origin; and in the third, defcribes the means of deftroying them.

Inteftinal Worms, according to our author, conftitute a particular clafs in the animal kingdom, which in the Linnean fyftem, might very properly be placed next to that of *teftacea.* Thefe worms, he obferves, are, in general, either flat or round. Of the former of thefe he diftinguifhes three genera, viz. the *Ligula*, *Fafciola*, and *Tænia.* The firft of thefe, the *Ligula*, is defcribed as a worm refembling a ribband, without articulations. The fpecies of this genus are, 1. the *Ligula pifcium*, and 2. the *Ligula avium.* The *Fafciola*, we are told, is diftinguifhed by its two fuckers, one at its extremity, and the other under its belly, by either of which it is able to fix itfelf. Two fpecies of this genus are defcribed, viz. 1. the *Fafciola hepatica* Linn.; and 2. the *Faciola Lucii* Muller.

In defcribing the *Tænia*, our author remarks, that its neck terminates in a little knob which forms the head of the worm, in which are four mouths or fuckers. Each articulation of the tænia, he obferves, has its ovary, and one or two tubes for depofiting the eggs. Thefe tubes have their origin in the ovary, and terminate at the the ſide of the worm. Each articulation is filled with a great number of eggs, which may be eaſily ſqueezed out, but he has not been able to ſatisfy himſelf as to the manner in which theſe eggs are fecundated.

Beſides the four mouths or ſuckers in the head of the tænia, Dr. Bloch has obſerved, that ſeveral ſpecies of this genus are furniſhed with a trunk which they are able to thruſt out or draw inwards; and on the abſence or preſence of this trunk, he has founded the ſpecific characters of his diviſions of this genus into *Tæniæ inarmatæ* and *Tæniæ armatæ*. Under the firſt of theſe diviſions we meet with ſixteen ſpecies, viz. 1. *Tænia lanceolata*; 2. *Tænia lanceolata nodoſa*; 3. *Tænia rectangulum*; 4. *Tænia articulis rotundis*; 5. *Tænia lineata*; 6. *Tænia villoſa*; 7. *Tænia articulis convideis*; 8. *Tænia collo longiſſimo*; 9. *Tænia cylindracea*; 10. *Tænia tenuis nodis inſtructa*; 11. *Tænia lævis*; 12. *Tænia capite truncato*; 13. *Tænia collari nigro*; 14. *Tænia vaſis nutriciis diſtinctis*; 15. *Tænia cucumerina*; 16. *Tænia lata hominis.* — Of the *Tæniæ armatæ*, the ſpecies are, 1. *Tænia tricuſpidata*; 2. *Tænia collo breviſſimo*; 3. *Tænia canina*; 4. *Tænia cucurbitina* (tænia folium *Linn.*)

The

The round worms are divided by our author into eight genera, the names of which, and of the different fpecies of each genus, are as follows, viz.

I. *Vermis veficularis.*

1. Vermis veficularis tæniæ formis.

2. ——— ——— eremita.

3. ——— ——— focialis.

II. *Echinorynchus.*

1. Echinorynchus gigas.
2. Echin. capite et collo armato.

III. *Afcaris inteftinalis.*

1. Afcaris lumbricoides.
2. ——— acus.
3. ——— vermicularis.
4. ——— papillofus.

IV. *Trichuris.*

V. *Gordius.*

1. Gordius inteftinalis.
2. ——— viviparus.
3. ——— Harangum.

VI. *Chariophyllus.*

VII. *Cuculanus.*

1. Cuculanus viviparus.
2. ——— convideus.

VIII. *Chaos inteftinalis.*

1. Hirudo inteftinalis.

2. Chaos inteftinalis cordiformis.

In the fecond fection of his work, the author endeavours to prove, that inteftinal worms are deftined by nature to be generated and to live in the bodies of other animals. Among other arguments in fupport of this opinion, he obferves, that they are found in the fœtufes of different animals in *utero*; that they refift the digeftive power of the ftomach; and that they conftantly die when expelled from, or taken out of the body. He has, indeed, been able to preferve a few fpecies in water or milk for the fpace of five or fix days, but none of them lived beyond that time. Several animals, he remarks, have fpecies of worms peculiar to themfelves, and which cannot live in any other. And as a farther proof to his doctrine, he adds, that they have no eyes; becaufe as the rays of light never reach them, they are in no need of vifual organs: and that they are without antennæ, becaufe, from their fituation, they can have no occafion to avoid danger.

Concerning the effects of worms in the animal body, Dr. Bloch obferves, that, in general, they become dangerous only when, from their bulk or number, they deprive the body of its due fhare of nutriment.

In the third fection, he treats of the means of deftroying thefe worms. The remedies he advifes for this purpofe are fuch as are generally recommended, in cafes of this fort, by the lateft and moft approved practical writers.

