# Errata

**Published:** 1785

**Authors:** 


					p. 77, 1. J tor JaciQla, read JaJctoLa. ?
p. 80, 1. 16, fcr of his doctrine, read oj this doctrine*

				

